# ERP Evidence for Inhibitory Control Deficits in Test-Anxious Individuals

**DOI:** 10.3389/fpsyt.2019.00645

**Published:** 2019-09-06

**Authors:** Wenpei Zhang, Alain De Beuckelaer, Lirong Chen, Renlai Zhou

**Affiliations:** ^1^Department of Psychology, Nanjing University, Nanjing, China; ^2^Department of Business Administration, School of Business, Anhui University of Technology, Maanshan, China; ^3^Institute for Management Research, Radboud University, Nijmegen, Netherlands; ^4^Department of Personnel Management, Work and Organizational Psychology, Ghent University, Ghent, Belgium; ^5^Key Laboratory for NeuroInformation of Ministry of Education, University of Electronic Science and Technology of China, Chengdu, China

**Keywords:** test anxiety, inhibitory control, emotional Stroop (ES), numerical Stroop (NS), ERPs

## Abstract

**Introduction:** Individuals with test anxiety [i.e., high test anxiety (HTA)] always treat tests/examinations as a potential threat. This cognitive mode impairs these individuals’ ability of inhibitory control and leads to a high level of anxiety. However, characterizing aspects of HTA’s impaired inhibitory control ability are unclear and need to be studied.

**Methods:** Forty-six participants were recruited and divided into a HTA (N = 26) and low test anxiety (LTA; i.e., healthy control; N = 20) group. Self-reports (Test Anxiety Scale, State-Trait Anxiety Inventory for negative emotions) were obtained. An emotional Stroop (ES) task and a numerical Stroop (NS) task, causing different types of interferences, were used for assessing the emotional and cognitive aspects of attentional control ability (behavioral data). Event-related brain potentials (ERPs) were registered to further assess processing stages related to different aspects of attentional control ability.

**Results:** Compared with the LTA group, the HTA group has inhibitory control deficits of both emotional (see ERP components P1-P2-N2 and P3) and cognitive (see ERP component P3) interference. Compared with the LTA group, the HTA doesn't have lower accuracy in neither ES nor NS but displays longer reaction times only in ES. Additionally, the HTA group’s ES results also show that (1) the degree of emotional interference indicates the level of an individual’s anxiety, and (2) the ERP component P2 may serve as an index of the level of test anxiety.

**Conclusion:** HTA individuals have extensive inhibitory deficits for both emotional and cognitive aspects; however, impairment impacts more on emotional aspects than on cognitive aspects. Additionally, as compared to NS, the negative impact of more impaired processing stages on task performance is more substantial in ES.

## Introduction

Test anxiety is a situation-specific form of anxiety disorder; individuals who suffer from this anxiety disorder tend to appraise performance evaluative situations (e.g., taking an exam) as threatening, and continue to be in high anxiety (1). Test anxiety manifests itself at the individual level in cognitive, affective-physiological, and behavioral characteristics (2). If the individual expects exam results to have great impact (e.g., on the course of his/her life), the exam is perceived as “threatening,” and the individual will display symptoms characterizing high anxiety (3). In most etiology models of anxiety disorders, cognitive evaluations of a (threatening) stimulus (i.e., a “cognitive pattern”) affects the etiology and the maintenance of anxiety in the individual (4–6).

The cognitive pattern may cause and maintain anxiety through affecting an individual’s ability of attentional inhibitory control. An individual who exerts inhibitory control is able to inhibit task-unrelated information or incorrect automatic responses when reacting to task-related information (7, 8). Worry caused by negative cognition stemming from an exam’s appraisal leads to anxiety and elevates the individual’s vulnerability to anxious cues. This vulnerability increases the degree of interference from exam/test-related information, which is unrelated to completing the task (i.e., task unrelated information), and decreases the ability of inhibitory control (9, 10). So, in comparison to low test anxiety (LTA) individuals, high test anxiety (HTA) individuals tend to show higher interference by test-related information than by neutral (i.e., including test-unrelated) information (11–13), an interference that may affect the allocation of attentional resources during the processing of task stimuli (13). More specifically, this interference may reduce the attentional resources used for the task at hand, impairing the individual’s task performance and increasing the level of anxiety in the individual (14, 15).

Attentional control theory assumes that anxiety impacts two dimensions of task performance: (performance) “effectiveness” and (processing) “efficiency” (5). Effectiveness refers to an individual’s “performance quality” usually measured as accuracy in task performance. Efficiency is conceptualized as the level of allocated resources used by an individual to process task stimuli, a conceptualization which is usually measured by the reaction time (RT) (5, 16). Most studies have shown that an inhibitory control deficit in HTA individuals led to detrimental efficiency, and sometimes even to detrimental effectiveness (13–15). Whether anxiety impairs the effectiveness may depend on cognitive load (17); when cognitive load is relatively low, anxious individuals apply some compensatory strategies, such as sacrificing on efficiency (causing the RTs to increase), to achieve equal effectiveness as non-anxious individuals [see, for instance, Refs. (18–21)].

However, previous evidence on these impairments all stem from emotion-activating experimental conditions in which participants needed to inhibit (test-related) threatening interference (11–13). In our view, it is necessary to make a conceptual distinction between (and also examine) different types of interference, namely, emotional and cognitive interference. Two reasons (explained below) make such distinctive conceptualization necessary.

*Reason no. 1:* Emotional and cognitive interference may reveal different cognitive aspects (i.e., the cognitive pattern) in HTA individuals. Two cognitive aspects are worthwhile mentioning. The first cognitive aspect concerns the impairment of emotional interference (such as test-related threatening interference), which is indicative of a negative cognitive mode related to test-related threatening information (13, 22, 23), including vigilance and further processing of threatening information (24); this first cognitive aspect manifests itself in both an early and a late stage of processing [e.g., Refs. (25, 26)]. The second cognitive aspect concerns the impairment of cognitive interference, which is indicative of an impaired cognitive function, attesting to a difficulty in inhibiting general interference (24); this second cognitive aspect manifests itself in a late stage of processing only (27, 28).

*Reason no. 2:* These two types of interference may lead to differential task performance. As impaired processing stages go hand in hand with a relatively high cognitive load (5), an impact on task performance is to be expected. The nature of the impact (on cognitive pattern, impaired processing stage, and task performance) is unclear at present. The differentially impaired processing stages involved in emotional and cognitive inhibition (as caused by different types of interference) may subsequently result in a differential impact on task performance.

In the present study, we relied on an emotional Stroop (ES) (29, 30) and a numerical Stroop (NS) (31, 32) paradigm to study the characteristics of inhibitory control ability when HTA individuals are exposed to test-related threatening interference and cognitive interference, respectively. In the Stroop paradigm, the stimulus, which is one word/number, reflects two dimensions at the time: (a) a task-related dimension, and (b) an interfering dimension. Consequently, one may systematically compare the time-course of an ongoing “competition” (for available attentional resources) between the two dimensions, and also assess how exactly different stages of processing are influenced by different types of interference (33). The ES paradigm is instrumental in studying vulnerability to threatening information, which is indicative of a negative mode of threats (34–36). Individuals are expected to display longer RTs and/or lower accuracy for threatening words, especially if they experience difficulties in inhibiting affective interference and, due to this inhibition, do not manage to recruit attentional resources (13, 37–39). The NS paradigm is instrumental in studying impairment of the cognitive inhibitory function. Individuals are expected to display longer RTs and/or lower accuracy for task-irrelevant aspects (for instance: the one-digit number that is larger in value is depicted in a smaller physical size), especially if they experience difficulties in inhibiting general interference (40–44).

Conventional Stroop studies are limited in that they only collect behavioral (i.e., mainly RT) data (45), while the use of event-related potentials (ERPs) in combination with behavioral data can provide many details on the stage in which the processing difficulties occur (46, 47). ERPs are known to have high temporal resolution, and therefore allow adequately “tracking” fast and temporal processing changes (48). Through the analysis of the “magnitude” of very specific ERP components (49), typically for different groups of stimuli (e.g., threatening and neutral stimuli), a researcher may collect clear indications of the nature of processing and processing difficulties encountered. For example, the magnitude of the ERP components P1, P2, and N2 are always examined when analyzing the automatic processing of emotional stimuli. The magnitudes of ERP components P1 and P2 indicate the allocation of attentional resources during the early (especially perceptual) stage of stimulus processing, an allocation that is indicative of the degree of hypervigilance of the stimulus (50–53). Additionally, the magnitude of ERP component N2 (especially when followed by perceptual components such as P1 and P2) indicates the automatic and facilitated processing following hypervigilance of the stimulus (50, 51). The magnitude of ERP component P3 is indicative of the cognitive processing of both emotional and cognitive stimuli; P3 is a component that reflects the allocation of attentional resources during a relatively late and cognitive stage of stimulus processing (44, 50–52). ERPs are known to change as a response to processing affective (i.e., emotional) stimulus content (rather than non-affective, that is, neutral stimulus content) (54–56). In addition, the magnitude and latency of ERP components adequately describe (processing) efficiency, and the activation of a (compensatory) strategy to reduce the effect of impaired efficiency on task performance (17, 28, 55). Therefore, ERPs offer very suitable descriptors of attention paid to stimuli/information in different processing stages, especially for HTA individuals as contrasted to LTA individuals (10, 48).

In sum, the purpose of the present study is to further examine how test-related threatening and cognitive interference impact inhibitory control deficits, processing efficiency, and task performance in HTA (as opposed to LTA) individuals. This examination is based on ES and NS paradigms, but relies, in addition to registering conventional behavioral outcomes (RTs), also on the registration and analysis of ERPs. As such, this study’s design may offer useful insights in the nature of stimulus processing under different conditions of interference. Based on our reasoning presented above, we predict that (a) HTA individuals have both emotional and cognitive inhibitory control deficits; the emotional inhibitory control deficit manifests itself in both the early and late processing stages, while the cognitive inhibitory control deficit manifests itself in the late processing stage only, and (b) for HTA individuals, the different processing stages involved in emotional and cognitive control deficit attest to a more severe impairment of task performance in ES than in NS.

## Methods and Materials

### Participants and Assignment to the LTA and the HTA Group

Individuals (from now on referred to as “participants”) were recruited through posters or online advertisements. Eighty-two students from Nanjing University signed up for participating in the experiments, and 46 students got eventually selected for participation in the study. According to conventional criteria set for high statistical power (57), we must conclude that our actual sample size combined with the effect sizes as reported in this study leads to high levels of statistical power, that is, the statistical power for each significant difference reported in our (repeated-measures) ANOVA is consistently higher than 98% [1-β > .98, α = .05, using the G-power software (58)]. Participants (aged from 18–26 years; all self-declared right handed) were chosen and assigned to a high test-anxious (HTA) group (n = 26; 12 males; 21.27 ± 1.89 years) and to a low test-anxious (LTA) group (n = 20; 9 males; 21.35 ± 2.96 years; not significantly different across groups: *t*_(1,44)_ = .113, *p* = .910) based on their score obtained with the (self-reported) Test Anxiety Scale survey instrument (TAS) (6). Thirty-six students were excluded because (a) they are neither HTA nor LTA, and/or (b) they appeared to have suffered or still suffer from currently known psychiatric disorders (e.g., depression, bipolar disorder, and substance abuse) as diagnosed (just before the start of this study) by a self-completed Structured Clinical Interview for DSM-IV (59). For descriptive purposes also the (self-reported) State-Trait Anxiety Inventory survey instrument (STAI) (60) was administered. STAI was used to unravel the effects of state and trait aspects of test anxiety on interference. Our study was carried out in accordance with a study protocol designed by ourselves, a study protocol that was approved by the Ethics Committee of Nanjing University, School of Social and Behavioral Sciences; the principles on the basis of which the Ethics Committee approves study protocols are described in a document entitled “Ethical Evaluation of Research Projects at the Department of Psychology, School for Social and Behavioral Sciences, Nanjing University.” All study participants gave written informed consent in accordance with the Declaration of Helsinki. In the next paragraphs, details on both survey instruments are given.

### Questionnaires

The Chinese version of TAS (6) was administered. The TAS contains 37 survey items reflecting symptoms of test anxiety, all of which are scored using the two true/false answer categories. A participant’s total score ranges from 0 to 37. The higher the participant’s total score, the more test anxiety symptoms are experienced. As documented in other studies (61), the Chinese version of TAS shows a Cronbach’s alpha coefficient of .64 and a test-retest reliability coefficient of.61. Participants are assigned to (a) the HTA group if their TAS total score exceeds or equals 20, and (b) assigned to the LTA group if their TAS total score falls below or equals 12 (61, 62).

The Chinese version of the State-Trait Anxiety Inventory (STAI) was administered (60). The STAI contains 40 survey items, all of which are scored on a four-point, Likert type scale ranging from 1 (not at all) to 4 (very much so). Twenty survey items measure state anxiety (S-STAI) and the other 20 items measure trait anxiety (T-STAI). Obviously, the higher the S-STAI/T-STAI total score, the more symptoms of state-/trait- anxiety are observed, respectively. As documented by Li and Qian (60), the Chinese STAI scale shows a Cronbach’s alpha coefficient of .91 for S-STAI and .88 for T-STAI (60). In contrast to TAS, no assessments of test-retest reliability have been published for STAI.

### Stimuli, Behavioral Paradigm, and Test Procedure

Participants showed up in the laboratory to participate in the experiment within 1 week after completing TAS and STAI. Participants were connected to a 64-electrode head cap (technical details are given further on) and instructed to relax for 2 min. Then, they either started with the ES or the NS experiment (determined at random) and, after completion, went on with “the other” experiment. Both the ES and NS experiment consisted of a “practice block” (not providing data for analysis) and, subsequently, an “experimental block” (providing data for analysis). When participating in the ES and NS, participants were always asked to finish the tasks “as quickly and accurately as possible.” After completion of both the ES and NS experiment, the electrode head cap was removed and participants got paid the equivalent of about 6 US dollars (i.e., 40 Renminbi).

#### Emotional Stroop (ES)

The ES task design was similar to that of Thomas et al. (52). Participants were asked to name the color of the word displayed (on screen) regardless of the word’s meaning. The stimuli (words) chosen constitute two experimental conditions (or experimental groups): test-related threatening (e.g., “test paper” and “score”) words and neutral (e.g., “garden” and “shoes”) words. Each experimental condition includes 15 words that were selected based on scores (see **Table S1**) from a pool of 30 potential neutral/test-related threatening words that were scored by 40 individuals who did not participate in the present study. Words ranged in length from two to four Chinese characters. The words subtended a maximum of 3.81 degrees of visual angle in width. The words subtended 1.27 degrees in height.

Test-related threatening words and neutral words were picked out by following a procedure similar to that of Thomas et al. (52); in particular, they were picked out according to their threatening degree (by asking “How threatening is this word to you [including related unpleasant thoughts and feelings, such as worry or/and anxiety]?”) and test-related degree (by asking “How relevant is this word to “test”)?, and matched for frequency of usage (by asking “How often do you use or see this word?”). A seven point Likert-type scale ranging from 1 (not at all) to 7 (severely threatening/strongly relevant/always, respectively) was used. A significant difference exists in the threatening degree (*t*_(28)_ = 30.190, *p* < .001) and the test-related degree (*t*_(28)_ = 38.166, *p* < .001) between the two groups of words, of course in the expected direction, that is “threatening” words are relatively more threatening and test-related. As far as frequency of usage is concerned, no significant difference exists between the group of threatening words and the group of neutral words (*t*_(28)_ = 1.436, *p* = .162).

Two experimental blocks were offered: (a) a practice block, and, afterwards, (b) an experimental block. The practice block contained six trials; the set-up of the practice block is analogous to the experimental block, except for (a) the number of words offered (6 rather than 120 words), and (b) the fact that all words were neutral. All six words offered in the practice block were not used in the experimental block. To enable participants’ learning in the practice block, the message “correct” or “false” was given after each trial (obviously, such message was not given in the experimental block). The experimental block contained 120 trials (every word was offered four times, picked randomly, and the word was displayed 50% of the times in red, and 50% of the times in blue). Each trial started with a fixation point “+” on a computer screen; that fixation point stayed on screen for 200 ms. Next, the screen turned blank and lasted for a time in-between 800 and 1200 ms (duration was randomly picked). Subsequently, the word (randomly picked) was presented in the center of the screen against a white background. This trial ended when (a) the participant reacted (i.e., pushed a button to indicate the color of the word) or (b) failed to react within 2000 ms. In-between consecutive trials, a screen with a white background was shown for a time in-between 1000 and 2000 ms (duration was randomly picked).

#### Numerical Stroop (NS)

The NS task design was similar to that of previous studies (63, 64). Participants were asked to compare the numerical value (i.e., indicate the larger value) of a pair of two white one-digit numbers positioned left and right against a gray background. In order to reduce the impact of distance in value, one digit was always three units larger in value than the other (1-4 or 4-1; 2-5 or 5-2; 3-6 or 6-3, etcetera) in each pair (65). Which position (left or right) was larger in value was determined at random. The pairs of one-digit numbers constituted three experimental conditions: (a) congruent, (b) incongruent, and (c) neutral. In a congruent pair, the one-digit number showing the larger numerical value was also the larger one in physical size (200 points in physical size and the other one-digit number 140 points in physical size). In an incongruent pair, the one-digit number showing the larger numerical value was the smaller one in physical size, that is, 140 points in physical size, whereas the other one-digit number was 200 points in physical size. In a neutral pair, two digits were of the same physical size (half of the times 140 points and half of the times 200 points). The words subtended almost 3.81 degrees of visual angle in width. The words subtended 1.59 degrees for larger physical size number in height and 0.5 degrees for smaller physical size number in height.

Two experimental blocks were offered: (a) a practice block afterwards, and (b) an experimental block. The practice block contained six trials; the set-up of the practice block is analogous to the experimental block, except for the fact that in the practice block only neutral pairs are offered. Just as in ES, participants were given feedback (correct/false) after each trial in the practice block (but not in the experimental block). The experimental block contained 108 trials (each experimental condition consisted of 36 trials). Each trial started with a fixation point “+” on a computer screen; that fixation point stayed on screen for 200 ms. Next, the screen turned blank and lasted for a time in-between 800 and 1200 ms (duration was randomly picked). Subsequently, the number pair (randomly picked) was presented in the center of the screen against a gray background. This trial ended when the participant gave a reaction (pushing a button to indicate which one-digit number was the larger one in value) or failed to react within 5000 ms. In-between consecutive trials, a screen with a gray background was shown for a time in-between 1000 and 2000 ms (duration was randomly picked).

### Electrophysiological Recording and Analysis

A NeuroScan recording system and a 64-electrode head cap designed according to the International 10/20 system formed the technical equipment to collect electrophysiological (EEG) data. The electrode placed on the left mastoid served as a reference during recording. A horizontal electrooculogram (HEOG) was derived from EEG data collected by electrodes placed on the outer canthi of the eyes, and a vertical electrooculogram (VEOG) was derived from EEG data collected by an electrode placed above and below the left eye. The impedances were kept below 5 kOhm. EEG was recorded by a DC model with a sampling rate of 1000 Hz.

Offline analysis of EEG data was enabled through the software “Curry 7.0.8.” EEG data was re-referenced to the average of the left and right mastoids, filtered using a 30 Hz bandwidth (24 dB/octave slope), and corrected for ocular artifacts. To retrieve ERPs from participants’ responses to stimuli, data were epoched from 200 ms pre-stimulus to 1,000 ms post-stimulus, baseline-corrected by 200 ms pre-stimulus, and averaged for all experimental conditions. Interest areas were defined based on two steps. First, the interest areas of related ERP components from previous studies were identified to select specific ERP components for use in the present study; in ES: ERP components P1, P2, N2, and P3 components were selected (50–53); in NS: ERP component P3 was selected (44). Second, in the present study, the exact interest areas of each ERP component were identified based on the grand average latency (i.e., the average time intervals between stimulus onset and peak of each condition for each experimental group) determining the middle point of the interest area. Eventually, time windows of each ERP component were, in ES, P1 (120–170 ms), P2 (210–260 ms), N2 (240–290 ms), and P3 (320–370 ms) and, in NS, P3 (neutral: 300–400 ms; congruent: 300–350 ms; incongruent: 420–470 ms). ERP components’ data were analyzed at five electrodes (Fz, FCz, Cz, CPz, and Pz). Mean amplitudes (i.e., mean amplitude of the peaks) and latency of each ERP component were calculated for each condition and for each participant. Per electrode (measuring a component) data on 60 trials in ES and 36 trials in NS were collected for each condition.

### Statistical Analysis

TAS and STAI data (i.e., self-reported data) were analyzed using (standard) independent samples t-tests. In agreement with other ERP studies on attention [e.g., Refs. (8, 66)], ES and NS data from (a) erroneous trials (i.e., false answers) and (b) trials with RTs exceeding three standard deviations from the participant’s mean RT (as calculated for the experimental condition) were removed prior to analysis. Also in agreement with previous studies (11, 67–69), statistical analyses mainly aimed at examining the statistical significance of between-group (i.e., HTA versus LTA) differences in attentional control ability as observed in given (experimental) conditions, rather than between-condition differences in attentional control ability for HTA individuals or, alternatively, LTA individuals. For behavioral and ERP data, in ES, RT (in milliseconds), accuracy (percent correct, %), and ERP amplitudes data were analyzed using a 2 group (HTA, LTA) × 2 condition (test-related threatening, neutral) repeated-measures ANOVA. In NS, RT, accuracy, and ERP amplitudes data were analyzed using a 2 group (HTA, LTA) × 3 condition (neutral, congruent, incongruent) repeated-measures ANOVA. Both behavioral and ERP data were corrected according to the Greenhouse–Geisser correction after running the ANOVAs. In our correlation analysis, we relied on the Pearson correlation. In all significance testing, we relied on the conventional criterion of *p* < .05. Significance is reported by reporting the exact significance (format used: “*p* = …”), unless the p-value is less than.001 (format used: “*p* < .001”).

## Results

### TAS and STAI Results

In the present study, TAS shows a Cronbach’s alpha coefficient of 0.87 and a test-retest reliability coefficient of 0.74, and STAI shows a Cronbach’s alpha coefficient of 0.95 for S-STAI and 0.90 for T-STAI. Descriptive statistics (on TAS and STAI) for both HTA and LTA individuals are shown in **Table 1**. In line with the expectations, the HTA group displays a higher degree of test anxiety, state anxiety, and trait anxiety than the LTA group.

**Table 1:** The TAS and STAI subscales scores in the HTA and LTA group (M ± SD).

	HTA (N = 26)	LTA (N = 20)	*t*	*p*
TAS	27.85 ± 4.78	8.65 ± 2.76	17.11	<.001
S-STAI	56.77 ± 9.28	31.55 ± 4.85	11.91	<.001
T-STAI	56.46 ± 9.19	31.95 ± 5.11	11.48	<.001

*HTA, high test-anxious; LTA, low test-anxious; TAS, Test Anxiety Scale survey instrument; S-STAI, state subscale of State-Trait Anxiety Inventory; T-STAI, trait subscale of State-Trait Anxiety Inventory.*

### Behavioral Results

#### Behavioral Results in ES

Mean RTs, accuracy, and stimulus types are shown in **Table 2**. RT data show a significant condition main effect (*F*_(1,44)_ = 6.005, *p* = .018, *η* *^2^* = .120) and a significant group × condition interaction effect (*F*_(1,44)_ = 4.447, *p* = .041, *η* *^2^* = .092). No significant group main effect was found (*F*_(1,44)_ = 2.323, *p* = .135, *η* *^2^* = .050). Additional simple effect analysis shows that, in contrast to the LTA group (*F*_(1,44)_ = .052, *p* = .821, *η* *^2^* = .001), the HTA group has a longer RT for test-related threatening words than for neutral words (*F*_(1,44)_ = 11.953, *p* < .001, *η* *^2^* = .214).

**Table 2:** Emotional Stroop results on the HTA and LTA group: (1) reaction times (RTs, in ms) of test-related threatening words, neutral words, and test-related threatening interference, and (2) accuracy (in %) of test-related threatening and neutral words (M ± SD).

Condition	RT		Accuracy	
	HTA (N = 26)	LTA (N = 20)	HTA (N = 26)	LTA (N = 20)
Test-related threatening	502.78 ± 95.13***	453.90 ± 78.47	99.88 ± 1.45**	98.35 ± 1.63
Neutral	480.84 ± 89.91	452.25 ± 79.58	97.88 ± 2.17	99.25 ± 0.96*
Interference	21.94 ± 38.77	1.64 ± 21.14		

*p < .05, **p < .01, ***p < .001.

*HTA, high test-anxious; LTA, low test-anxious; Interference: the RT as observed in the test-related threatening condition minus the RT as observed in the neutral condition.*

Accuracy data show a significant condition main effect (*F*_(1,44)_ = .038, *p* = .846, *η* *^2^* = .001) and a significant group × condition interaction effect (*F*_(1,44)_ = 13.833, *p* < .001, *η* *^2^* = .239). No significant group main effect was found (*F*_(1,44)_ = .982, *p* = .327, *η* *^2^* = .022). Additional simple effect analysis shows that the LTA group has higher accuracy for neutral words than for test-related threatening words (*F*_(1,44)_ = 5.492, *p* = .024, *η* *^2^* = .111); on the contrary, the HTA group has higher accuracy for test-related threatening words than for neutral words (*F*_(1,44)_ = 8.814, *p* = .005, *η* *^2^* = .167).

A substantial correlation between behavioral data and TAS or STAI scores was found; in particular, a higher accuracy for test-related threatening words is associated with a higher T-STAI total score (*r* = .344, *p* = .019).

#### Behavioral Results in NS

Mean RTs, accuracy, and stimulus types are shown in **Table 3**. RT data show a significant condition main effect (*F*_(1,43)_ = 90.210, *p* < .001, *η* *^2^* = .672) and a significant group × condition interaction effect (*F*_(1,43)_ = 5.435, *p* = .011, *η* *^2^* = .110). No significant group main effect was found (*F*_(1,43)_ = 1.165, *p* = .286, *η* *^2^* = .026). Additional simple effect analysis shows that (a) for each group, significant RT differences exist between the three conditions (HTA: *F*_(1,43)_ = 24.225, *p* < .001, *η* *^2^* = .530; LTA: *F*_(1,43)_ = 39.288, *p* < .001, *η* *^2^* = .646) and (b) for each condition, no significant RT difference is observed between the HTA and the LTA group (Neutral: *F*_(1,44)_ = 1.776, *p* = .090, *η* *^2^* = .039, Congruent: *F*_(1,44)_ = 2.294, *p* = .137, *η* *^2^* = .050, Incongruent: *F*_(1,44)_ = .066, *p* = .799, *η* *^2^* = .001).

**Table 3:** Numerical Stroop results on the HTA and LTA group: (1) reaction times (RTs, in ms) and (2) accuracy (in %) in three experimental conditions (M ± SD).

Condition	RT		Accuracy	
	HTA (N = 26)	LTA (N = 20)	HTA (N = 26)	LTA (N = 20)
Neutral	504.43 ± 110.11	469.18 ± 48.60	99.54 ± 1.39	99.85 ± 0.67
Congruent	474.21 ± 99.55	437.62 ± 47.28	99.77 ± 0.81	99.85 ± 0.67
Incongruent	529.04 ± 99.27	522.97 ± 41.82	95.38 ± 5.73	95.00 ± 4.76
Congruent interference	−30.22 ± 26.42	−31.56 ± 25.99		
Incongruent interference	24.61 ± 41.82	53.79 ± 22.34		

*HTA, high test-anxious; LTA, low test-anxious; Congruent interference: the RT as observed in the congruent condition minus the RT as observed in the neutral condition; Incongruent interference: the RT as observed in the incongruent condition minus the RT as observed in the neutral condition.*

Accuracy results show a significant condition main effect (*F*_(1,43)_ = 31.054, *p* < .001, *η* *^2^* = .414). No significant group × condition interaction effect (*F*_(1,43)_ = .141, *p* = .729, *η* *^2^* = .003) and group main effect (*F*_(1,43)_ < .001, *p* = .996, *η* *^2^* < .001) is reported.

No substantial correlations between behavioral data and TAS or STAI scores were found.

### ERP Results

#### ERP Results in ES

**Figure 1:**
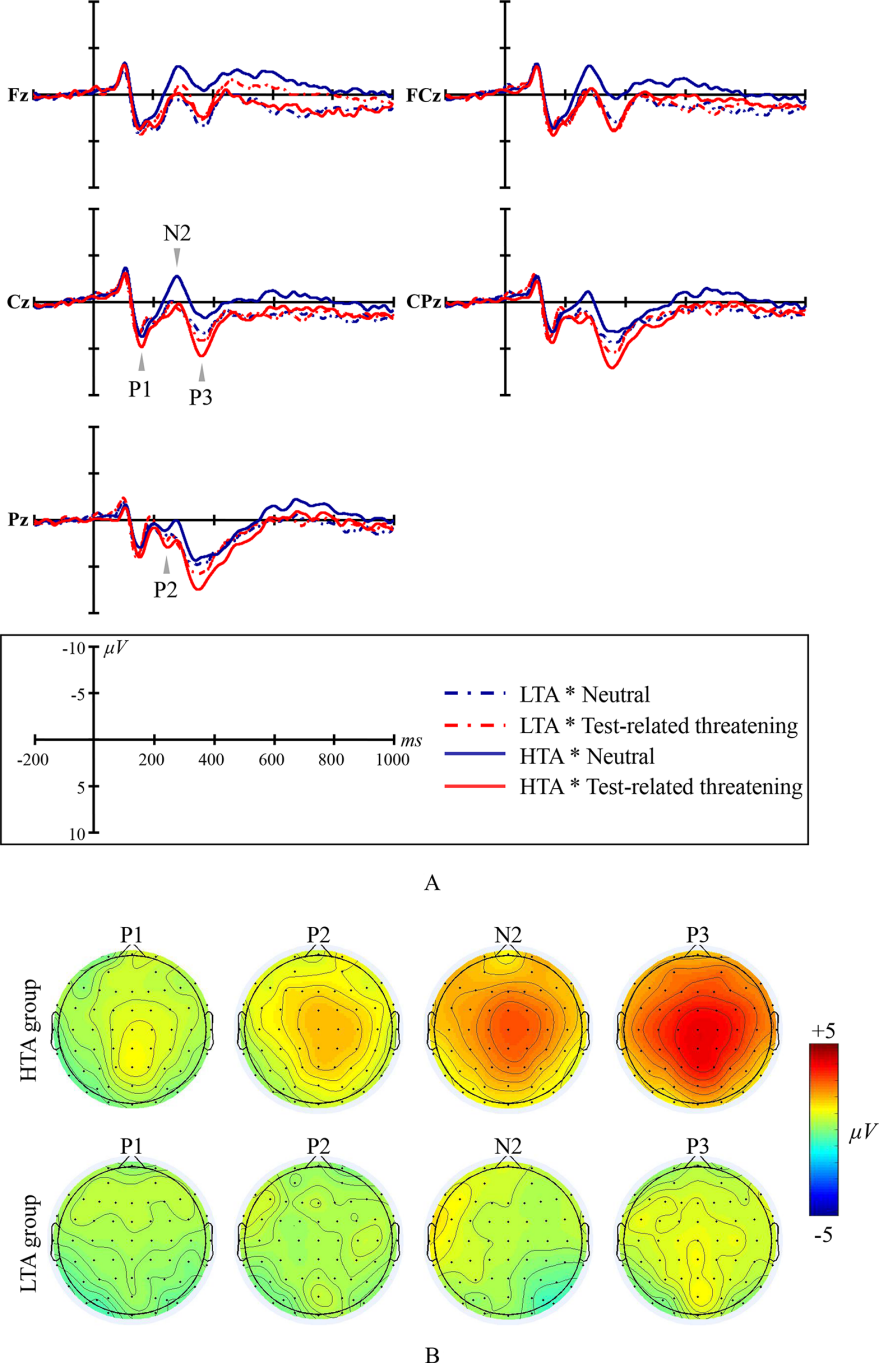
Emotional Stroop ERP waveforms and scalp topographic maps for both the high test-anxious (HTA) and the low test-anxious (LTA) group: **(A)** grand average ERPs elicited by test-related threatening and neutral words for the HTA and the LTA group at Fz, FCz, Cz, CPz, and Pz sites; P1, P2, and P3 are all registered at site Cz, CPz, and Pz; N2 was registered at site Fz, FCz, and Cz; **(B)** topographic distribution of the amplitude at the peak latency of the P1, P2, N2, and P3 difference waveforms (i.e., the amplitude emerging from exposure to test-related threatening words minus the amplitude emerging from exposure to neutral words) for both the HTA and the LTA group.

Grand average ERP waveforms and scalp topographic maps for ES are shown in **Figure 1**.

The amplitude of ERP component P1 shows a significant condition main effect (*F*_(1,44)_ = 4.989, *p* = .031, *η* *^2^* = .102) and a significant group × condition interaction effect (*F*_(1,44)_ = 4.928, *p* = .032, *η* *^2^* = .101). No significant group main effect was found (*F*_(1,44)_ = 2.74, *p* = .105, *η* *^2^* = .059; at site Pz). Additional simple effect analysis shows that, in contrast to the LTA group (*F*_(1,44)_ < .001, *p* = .993, *η* *^2^* < .001), the HTA group has a larger P1 amplitude for test-related threatening words than for neutral words (*F*_(1,44)_ = 11.405, *p* = .002, *η* *^2^* = .206).

The amplitude of ERP component P2 shows a significant condition main effect (*F*_(1,44)_ = 12.696, *p* = .001, *η* *^2^* = .224) and a significant group × condition interaction effect (*F*_(1,44)_ = 5.384, *p* = .025, *η* *^2^* = .109). No significant group main effect was found (*F*_(1,44)_ = .013, *p* = .909, *η* *^2^* < .001; at site Cz, CPz, and Pz, and take site Pz as an example for presenting significant results). Additional simple effect analysis shows that, in contrast to the LTA group (*F*_(1,44)_ = .683, *p* = .413, *η* *^2^* = .015), the HTA group has a larger P2 amplitude for test-related threatening words than for neutral words (*F*_(1,44)_ = 19.903, *p* < .001, *η* *^2^* = .311).

The amplitude of ERP component N2 shows a significant condition main effect (*F*_(1,44)_ = 24.378, *p* < .001, *η* *^2^* = .357) and a significant group × condition interaction effect (*F*_(1,44)_ = 15.989, *p* < .001, *η* *^2^* = .267). No significant group main effect was found (*F*_(1,44) = ._817, *p* = .370, *η* *^2^* = .018; at site Fz, FCz, and Cz, and take site Cz as an example for presenting significant results). Additional simple effect analysis shows that, in contrast to the LTA group (*F*_(1,44)_ = .390, *p* = .536, *η* *^2^* = .009), the HTA group has a smaller N2 amplitude for test-related threatening words than for neutral words (*F*_(1,44)_ = 45.916, *p* < .001, *η* *^2^* = .511).

The amplitude of ERP component P3 shows a significant condition main effect (*F*_(1,44)_ = 64.656, *p* < .001, *η* *^2^* = .595) and a significant group × condition interaction effect (*F*_(1,44)_ = 27.817, *p* < .001, *η* *^2^* = .387). No significant group main effect was found (*F*_(1,44)_ = .320, *p* = .574, *η* *^2^* = .007; at site Cz and Pz, and take site Pz as an example for presenting significant results). Additional simple effect analysis shows that, in contrast to the LTA group (*F*_(1,44)_ = 3.386, *p* = .073, *η* *^2^* = .071), the HTA group has a larger P3 amplitude for test-related threatening words than for neutral words (*F*_(1,44)_ = 101.943, *p* < .001, *η* *^2^* = .699).

No significant results are reported for the latency of the ERP components P1, P2, N2, and P3 (all *Fs* < 1.126, all *ps* > .335, all *η* *^2^* *s* < .06).

**Figure 2:**
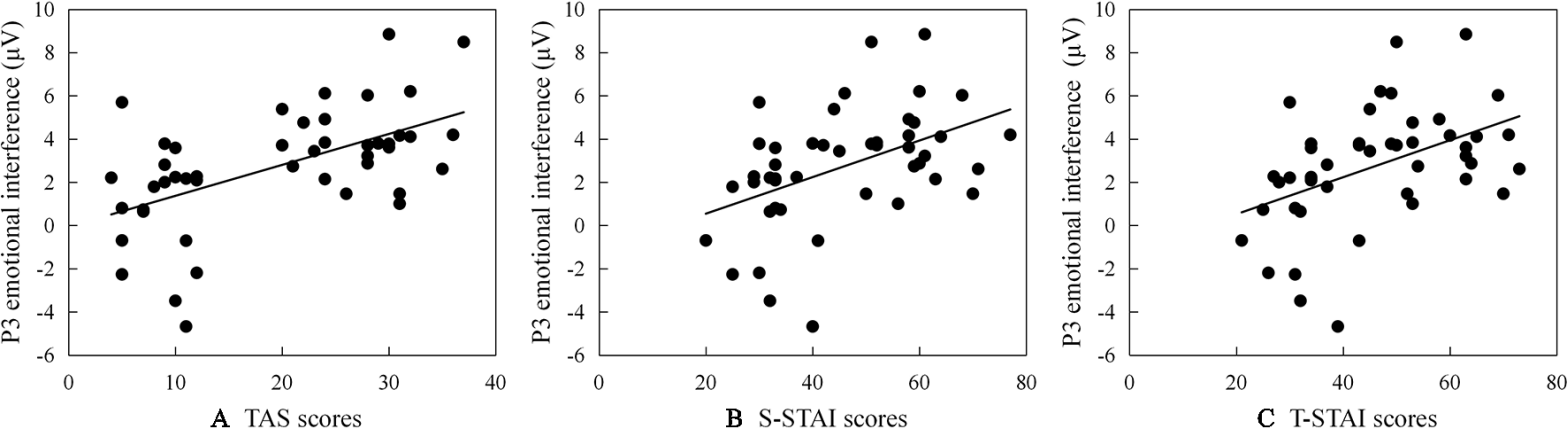
Scatter plots (including the best fitting linear regression line) describing correlations between P3 emotional interference (ERP amplitude as registered for the test-related threatening condition minus ERP amplitude as registered for the neutral condition) and **(A)** scores on the Test Anxiety Scale survey instrument (TAS), **(B)** scores on the State subscale of State-Trait Anxiety Inventory (S-STAI), and **(C)** scores on the Trait subscale of State-Trait Anxiety Inventory (T-STAI), respectively.

Significant correlations between ERP emotional interference (ERP amplitude as registered for the test-related threatening condition minus ERP amplitude as registered for the neutral condition) and TAS or STAI subscales scores are reported (see scatter plots in **Figure 2**): (a) larger P2 emotional interference is associated with a higher TAS total score (*r* = .377, *p* = .010); (b) larger N2 emotional interference is associated with a higher TAS and a higher STAI subscale score (for TAS: *r* = .366, *p* = .012; for STAI: *r* *_S-STAI_* = .343, *p* *_S-STAI_* = .020; *r* *_T-STAI_* = .384, *p* *_T-STAI_* = .008); and (c) larger P3 emotional interference is associated with a higher TAS total score and a higher STAI subscale score (for TAS: *r* = .550, *p* < .001; for STAI: *r* *_S-STAI_* = .554, *p* *_S-STAI_* < .001; *r* *_T-STAI_* = .546, *p* *_T-STAI_* = < .001).

#### ERP Results in NS

**Figure 3:**
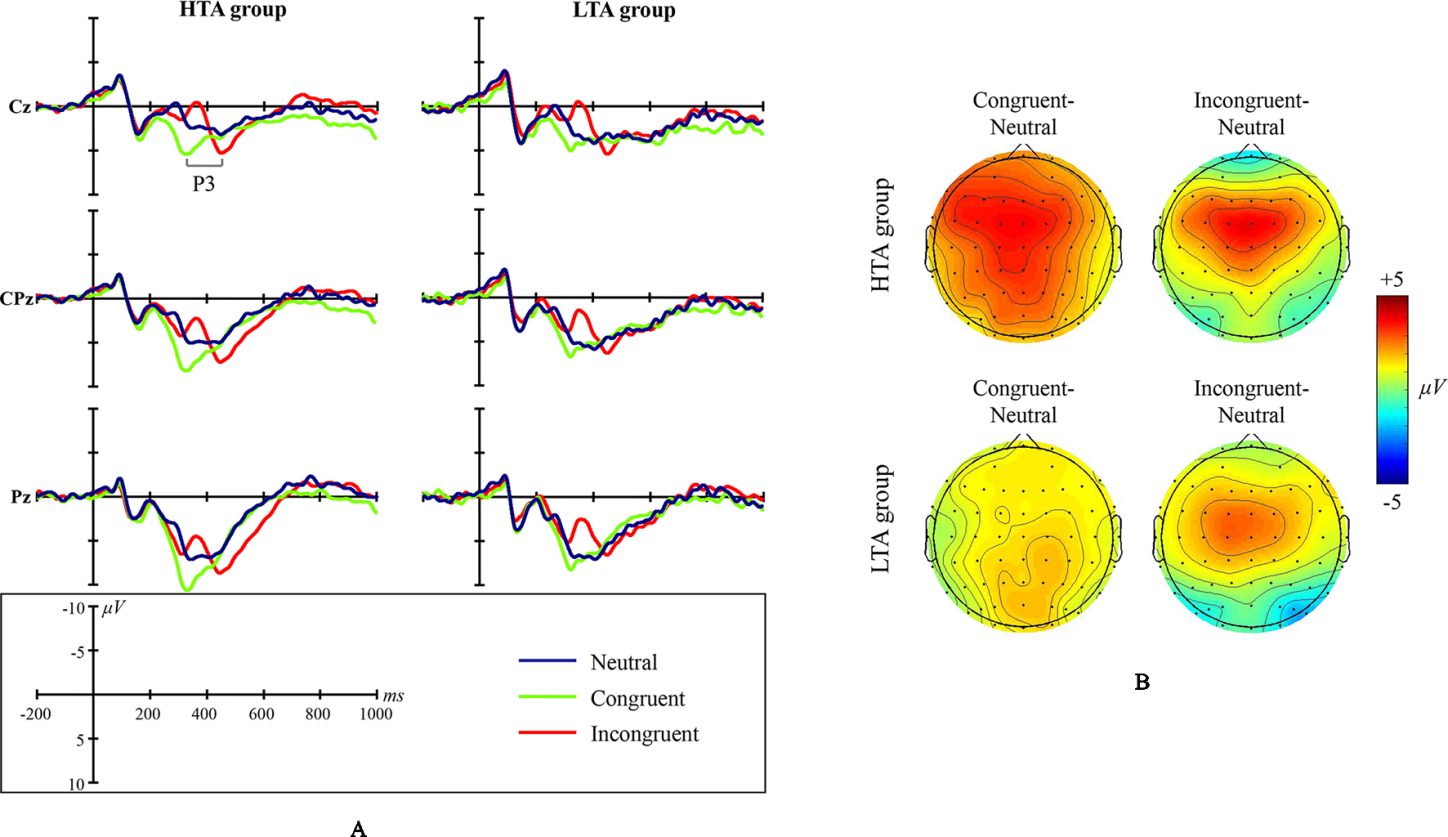
Numerical Stroop ERP waveforms and scalp topographic maps for both the high test-anxious (HTA) and the low test-anxious (LTA) group: **(A)** grand average ERPs elicited by the three experimental conditions (neutral, congruent, and incongruent); the P3 component was registered at Cz, CPz, and Pz sites. **(B)** topographic distribution of the amplitude at the peak latency of the P3 difference waveforms (i.e., the amplitude emerging from exposure to the congruent condition minus the amplitude emerging from exposure to the neutral condition, and the amplitude emerging from exposure to the incongruent condition minus the amplitude emerging from exposure to the neutral condition) for both the HTA and the LTA group.

Grand average ERP waveforms and scalp topographic maps for NS are shown in **Figure 3**.

The amplitude of ERP component P3 shows a significant condition main effect (*F*_(1,44)_ = 57.740, *p* < .001, *η* *^2^* = .568) and a significant group × condition interaction effect (*F*_(1,43)_ = 3.702, *p* = .033, *η* *^2^* = .147). No significant group main effect was found (*F*_(1,43)_ = .673, *p* = .417, *η* *^2^* = .015; site CPz). Additional simple effect analysis shows that (a) in contrast to the LTA group, the HTA group has a different P3 amplitude for three conditions (*F*_(1,43)_ = 18.929, *p* < .001, *η* *^2^* = .468), and (b) in contrast to the neutral condition, the HTA group has a larger P3 amplitude for the congruent condition (*p* < .001) and the incongruent condition (*p* = .004).

The latency of ERP component P3 shows a significant condition main effect (*F*_(1,43)_ = 86.342, *p* < .001, *η* *^2^* = .662) and a significant group × condition interaction effect (*F*_(1,43)_ = 3.784, *p* = .038, *η* *^2^* = .079). No significant group main effect was found (*F*_(1,43)_ = .142, *p* = .708, *η* *^2^* = .003). Additional simple effect analysis shows that for each experimental condition, no significant ERP difference exists between the LTA and the HTA group (Neutral: *F*_(1,44)_ = .433, *p* = .514, *η* *^2^* = .010; Congruent: *F*_(1,44)_ = .510, *p* = .479, *η* *^2^* = .011; Incongruent: *F*_(1,44)_ = .106, *p* = .746, *η* *^2^* = .002).

## Discussion

Considering both the behavioral and ERP results we suggest that, compared to LTA individuals, HTA individuals show deficits in inhibitory control and show different behavioral and ERP characteristics for different aspects of attentional inhibitory control. For test-related threatening interference, inhibitory deficit is observed in HTA individuals in both the perceptual and the cognitive processing stage, while for cognitive interference, inhibitory deficit is observed in HTA individuals only in the cognitive processing stage. This stage-related difference between two types of interference attest to the existence of different task performance in HTA individuals; more specifically, HTA individuals show more aspects of impaired task performance with lower efficiency (i.e., longer RTs) for test-related threatening interference than that for cognitive interference. Also, having observed no impaired effectiveness of task performance (i.e., accuracy), we suggest that HTA individuals apply a cautious strategy to complete the task in situations in which task performance could be impaired by inhibitory control deficits. Additionally, because of meaningful correlations between questionnaire scales’ scores and ES results, we present the results on questionnaire scales’ scores after the presentation of ES results.

### ES Results

In ES, longer RTs for test-related threatening words indicate that (a) HTA individuals show an inhibitory control deficit if they are exposed to a test-related threatening interference [see similar evidence, (11–13)] and (b) HTA individuals allocate extra attentional resources to process the threatening information instead of focusing on the task at hand. Additionally, ERP results further indicate that attentional resources are extra allocated in the early and late processing stages.

#### The Early/Perceptual Processing Stage

Through the study of characteristics of early/perceptual processing, one may appreciate the cognitive mode of emotional disorders. When individuals treat tests/examinations as threatening (i.e., are HTA individuals), emotionally congruent information is (i.e. test-related threatening words are) preferentially processed (70); preferential processing of information implies that the information is easily detected (in the perceptual stage) and processed prior to other information that is presented at the very same time. As the meaning of the word (i.e., the stimulus in ES Stroop) is irrelevant to the experimental task at hand, preferential processing of the emotional significance of the word should be conceived as an extra consumption of attentional resources of the central executive system and makes the nature of processing stimulus-driven rather than goal-driven (11, 18).

In our ERP results related to ES, the larger P1, P2, and smaller N2 amplitudes for test-related threatening words (in comparison to neutral words) in HTA individuals are indicative of a stimulus-driven processing in those individuals. Specifically, the ERP component P1 is known to be sensitive to (i.e., captures) emotional cues (71, 72), and the amplitudes of P1 may reveal hypervigilance (73) and one’s orientation to the information (74). If the information has negative meaning to the individual, a situation may occur in which the individual could easily detect this negative information (i.e., hypervigilance) and allocate extra attentional resources to rapidly orient to this negative information in the perceptual stage; typical in such situation is that P1 amplitudes are increased. The ERP component P2 captures both attention towards stimuli with negative meaning as well as inhibition of the interference (53, 75). If the negative information activates hypervigilance and occupies additional resources of the individual, the individual may attempt to inhibit the interference caused by the processing of this negative information and focus on the task at hand. If making such attempt is too difficult or simply unsuccessful (e.g., because of an inhibitory control deficit), the individual will continue to pay extra attention on processing the negative information, as indicated by increasing P2 amplitudes (52, 76). The ERP component N2 captures changes in attention automatically paid to certain information content (77, 78). Previous studies claimed that early ERP components jointly (e.g., P1, P2, and N2) capture perceptual processing and low-level attentional allocation (79, 80). Relatively small N2 amplitudes followed by relatively large P1 and P2 amplitudes would indicate that both automatic and facilitated processing of negative information are manifest (81, 82). The automatic nature of stimulus-driven processing reflects the cognitive schemata of HTA individuals in which tests/examinations are automatically treated as threatening (Beck’s theory) (83).

#### The Late/Cognitive Processing Stage

The larger P3 amplitudes for test-related threatening words (in comparison to neutral words) indicate that, in the late/cognitive processing stage, HTA individuals engage in top-down and elaborate processing of threatening stimulus content (78, 84, 85). The parallel distributed processing network model of ES by Mathews and Mackintosh (86) can provide a theoretical framework for understanding inhibitory deficits involved in early and late processing stages in HTA individuals. Information processed in the perceptual stage is monitored by the “threat assessment system.” The threat assessment system works as follows: when relevant threatening information (i.e., test-related threatening word) is presented to an HTA individual, the threatening information is encoded and marked by the threat assessment system; subsequently, semantic content and/or feelings of threat are activated. The way of processing in the early, perceptual stage may affect the way of processing in late, cognitive stage (43, 44). Once stimuli are intense enough, the threat assessment system will recruit attentional resources to process/assess stimulus content and respond to the task at hand. Intense stimuli cause individuals to consume more top-down attentional resources to process stimulus content, and exert greater efforts to inhibit interference.

Additionally, the level of emotional inhibitory control deficit relates to the level of anxiety. Specifically, (a) test-anxiety correlates substantially and positively with the degree of difficulty in inhibiting test-related threatening interference (difference of P2, N2, and P3 amplitudes between the threatening condition and the neutral condition). This positive correlation is in line with our ERP results. Compared to individuals with less symptoms of test anxiety (i.e., LTA individuals), HTA individuals are more strongly affected by test-related threatening stimulus content; in addition, the higher the threat caused by the stimulus, the easier it becomes for HTA individuals to process and detect stimulus content in the early, perceptual stage, followed by the more elaborate processing in the late processing stage; in sum, there are more attentional resources allocated to process the threatening stimulus; (b) the level of trait anxiety correlates substantially and positively with the degree of difficulty in inhibition (see similar results) (21, 87). Not completely unexpected, our study results also demonstrate that HTA individuals have a higher level of state and trait anxiety than LTA individuals. However, the difficulty of inhibition to threat is higher with higher levels of trait anxiety (but not higher with higher levels of state anxiety). Reasoning in a causal way, one could (cautiously) state that especially the trait aspects of test anxiety may impact the impairment of inhibition, whereas the state aspects of test anxiety have far less impact (88); (c) test-related threatening interference of P2 amplitudes is only found to be substantially correlated with TAS, and the correlation is found to be positive. As expressed before, difficulty in inhibition of processing threatening information in the early stage of processing is evident from the P2 component, and is known to be a characteristic element of the cognitive pattern associated with test anxiety (89, 90). This characteristic element signifies that test-related threatening information can be detected and cannot be inhibited. So, the P2 component may serve as a diagnostic for identifying test anxiety.

### NS Results

In NS, the behavioral results do not attest to an impairment of cognitive inhibitory control in HTA individuals. However, ERP results do provide evidence that HTA individuals display inhibitory control deficits for cognitive interference. Specifically, the larger P3 amplitudes for the congruent/incongruent condition (in comparison to the neutral condition) indicate that HTA individuals may have cognitive inhibitory control deficits, but such deficits were only encountered in the cognitive processing stage, a clear difference between the NS and ES results. This difference in impaired processing stages between ES and NS results may be an important reason for the difference in behavioral aspects of task performance (especially efficiency, that is, RT) between ES and NS results in HTA individuals. Specifically, the more impaired processing stages in ES (i.e., early and late processing stages), in comparison to NS (i.e., late processing stages only), the more cognitive load is involved in HTA individuals completing the tasks at hand (91). As indicated in the Introduction section, relative high cognitive load may lead to detrimental efficiency (13, 15), so the impaired behavioral aspect of task performance (i.e., longer RT) was observed in ES, but not in NS.

Additionally, in NS, the fact that no inhibitory control deficit was observed in the perceptual processing stage in HTA individuals may be because NS does not include emotional stimuli; as such, hypervigilance and related automatic processing were simply not activated. However, despite the fact that no inhibitory control deficit was observed in the perceptual processing stage, in NS, deficits in cognitive processing were still observed, indicating that HTA individuals may suffer from extensive inhibitory deficits (81). Particularly, HTA individuals were not only found to have difficulty in inhibiting the incongruent interference, an interference that negatively impacts task completion (see similar results) (92, 93), but were also found to have difficulty in inhibiting the congruent interference, an interference that facilitates a quick accomplishment of the task at hand. This difficulty in inhibiting the cognitive interference indicates that the extensive cognitive inhibitory deficits of HTA individuals are merely related to the appearance of interference rather than the exact nature (incongruent vs. congruent) of interference. Besides, we also observed a substantial difference in the latency of P3 among different conditions, a difference that is in line with RT differences observed across these different conditions. This difference in P3 latency may be due to varying difficulty in task completion across these different conditions, and this difference observed is also supported by previous studies (44, 94).

### Strategy for Dealing With Deficits in HTA

In contrast to an impaired efficiency observed in ES and NS observed in HTA individuals, no impaired effectiveness (i.e., accuracy) in ES and NS was observed in these individuals. As explained in the Introduction, HTA individuals may apply a “cautious strategy” to achieve satisfactory effectiveness (equal or better accuracy than LTA individuals) by decreasing efficiency (more attentional resources consumed or/and longer RT) (95). Additionally, accuracy of inhibition is known to be associated with the trait referred to as “emotion-driven impulsivity” (96). Individuals who are impulsive, increase the frequency of inhibitory errors (i.e., low accuracy) when they are in an intense emotional state (97, 98). Our study results demonstrate that, during the experiment, HTA individuals do not show behavior that typically characterizes the emotionally driven impulsive trait. When inhibiting interference in an ES and a NS task, HTA individuals make a comparable number of mistakes as LTA individuals, and, when processing threatening words rather than neutral words in ES, HTA individuals make fewer mistakes (i.e., have a higher level of effectiveness, that is, accuracy) than LTA individuals; moreover, for the ES task, a higher level of trait anxiety was found to correspond with fewer mistakes (i.e., higher accuracy), indicating that emotionally driven impulsivity is not a characteristic of HTA individuals when it comes to explaining task performance (measured as accuracy). Instead, HTA individuals who are in an anxious state tend to be “cautious” in that they avoid mistakes in completing the task. Being cautious in such state may, at least partially, explain why test anxiety does not always lead to impaired task performance (especially when assessed in terms of effectiveness; in other words, accuracy).

The present study has some limitations. We got some indications of (possible study restrictions due to) the occurrence of “ceiling effects” in ES and NS. More specifically, accuracy was very high for each condition (above 95%). The occurrence of ceiling effects may be the consequence of two limitations of our present study: (a) low task pressure (or the absence of high task pressure); alternatively, high task pressure (e.g., great importance of task performance) would allow for a better differentiation of participants’ task performance (e.g., accuracy) without imposing study restrictions due to the occurrence of ceiling effects (3). The participants in the present study were not subjected to high task pressure: given low task difficulty, participants could achieve high accuracy (99); and (b) highly educated participants: all HTA and LTA participants are highly educated, so these two groups may both have high accuracy, and this not being different in terms of accuracy (100). Future studies are required to further confirm (or partially disconfirm) our present study findings, for instance, by imposing higher task pressure and recruiting participants from a more heterogeneous (less highly educated) population.

In conclusion, HTA individuals show deficits of inhibitory control that can consume (additional) attentional resources without impairing accuracy. The inhibitory deficits reflect the etiological cognitive pattern of HTA individuals; during task completion and when confronted with interference (emotional or cognitive interference), HTA individuals recruit attentional resources to inhibit the interference. The deficits of inhibitory control can also appear in conditions (e.g., congruent interference) that are beneficial to accomplish the experimental task quickly. A crucial difference in the cognitive pattern of HTA individuals when confronted with emotional versus cognitive interference is that, in comparison to cognitive interference, emotional interference additionally affects the early processing stage and increases the required cognitive load. Thus, in comparison to LTA individuals, HTA individuals have lower behavioral efficiency of emotional interference as opposed to cognitive interference. Furthermore, considering the strong relationship between impaired attentional control ability and test anxiety, future studies aiming at diagnosing test anxiety could consider measuring attentional control ability by relying on the registration of important ERP components (such as P2 in this study) as an alternative method complementing or substituting the diagnosis of test anxiety through a (traditional) self-report questionnaire.

## Ethics Statement

This study was carried out in accordance with the recommendations of Ethical Evaluation of Research Projects at the Department of Psychology, School for Social and Behavioral Sciences, Nanjing University, Ethics Committee. The protocol was approved by the Ethics Committee. All subjects gave written informed consent in accordance with the Declaration of Helsinki.

## Author Contributions

RZ and WZ contributed the study structure. WZ and LC collected the experimental data. WZ wrote the first draft of the manuscript. RZ and AB made critical revisions. All authors approved the submitted version of this manuscript.

## Funding

This work was supported by grants from the Key Project of Philosophy and Social Science Research in Colleges and Universities in Jiangsu Province (2015JDXM001), the Research and Innovation Foundation of Academic Degree Graduate of Jiangsu Province (KYZZ16_0010), and the Humanity and Social Science Foundation of Education Committee of Anhui province (Key Program) (SK2017A0084).

## Conflict of Interest Statement

The authors declare that the research was conducted in the absence of any commercial or financial relationships that could be construed as a potential conflict of interest.
