# Supplementary material for: ERP Evidence for Inhibitory Control Deficits in Test-Anxious Individuals
Source: Front Psychiatry. 2019 Sep 6;10:645. doi: 10.3389/fpsyt.2019.00645 (PMC6743369; doi:10.3389/fpsyt.2019.00645)
Supplement: Supplementary file 1 [file Table_1.docx]

Supplementary Material

ERP Evidence for Inhibitory Control Deficits in Test-Anxious Individuals

Wenpei Zhang, Alain De Beuckelaer, Lirong Chen and Renlai Zhou

*** Correspondence:** Renlai Zhou: rlzhou@nju.edu.cn

# Supplementary Figures and Tables

## Supplementary Table

**Supplementary Table 1.** **The word stimuli in Emotional Stroop:** neutral and test-related threatening words were selected based on subjective ratings of threatening degree, test-related degree (comparison of the rate with a minimum or maximum norm), and frequency of usage (used for matching, that is “equalizing” both groups of words) on a seven point Likert-type scale ranging from 1 (not at all) to 7 (severely threatening/strongly relevant/always, respectively) (*M±SD*). The first fifteen words are neutral words and the last fifteen words are test-related threatening words.

| Word (translation) | Threatening degree | Test-related degree | Frequency of usage |
| --- | --- | --- | --- |
| 毛巾(Towel) | 2.23±0.66 | 2.28±0.68 | 6.28±0.68 |
| 房间(Room) | 2.35±0.62 | 2.53±0.60 | 6.18±0.78 |
| 布鞋(Shoes) | 2.38±0.77 | 2.38±0.70 | 6.33±0.57 |
| 椅子(Chair) | 2.45±0.60 | 2.73±0.72 | 6.08±0.80 |
| 鼠标(Mouse) | 2.38±0.70 | 2.63±0.74 | 6.33±0.69 |
| 运动鞋(Sports shoes) | 2.45±0.75 | 2.93±0.76 | 6.25±0.63 |
| 洗发水(Shampoo) | 2.28±0.82 | 2.73±0.75 | 6.25±0.74 |
| 自行车(Bicycle) | 2.33±0.73 | 2.75±1.01 | 6.15±0.83 |
| 方便面(Instant noodles) | 2.43±0.96 | 2.53±0.82 | 6.35±0.74 |
| 玻璃杯(Glass) | 2.48±0.91 | 2.80±0.85 | 6.28±0.75 |
| 公交车站(Bus stop) | 2.80±1.02 | 2.38±0.84 | 6.20±0.61 |
| 十字路口(Crossroads) | 2.83±1.08 | 2.75±0.78 | 6.28±0.75 |
| 街心花园(Street heart garden) | 2.78±0.97 | 2.95±0.71 | 6.15±0.58 |
| 塑料饭盒(Plastic lunch box) | 2.78±0.97 | 2.45±0.68 | 6.15±0.80 |
| 自选超市(Self-service supermarket) | 2.70±0.79 | 2.50±0.82 | 6.25±0.74 |
| 补考(Supplementary exam) | 5.13±1.26 | 5.88±0.94 | 6.43±0.64 |
| 答题(Answer) | 4.93±1.16 | 6.03±0.86 | 6.20±0.72 |
| 考场(Examination room) | 4.75±1.15 | 5.73±1.24 | 6.13±0.76 |
| 分数(Scores) | 4.83±1.22 | 5.78±1.14 | 6.25±0.71 |
| 考试(Examination) | 4.65±1.17 | 5.83±1.11 | 6.30±0.69 |
| 交卷子(Hand in an examination paper) | 4.85±1.58 | 5.55±1.08 | 6.20±0.79 |
| 考试题(Exam questions) | 4.70±1.30 | 5.63±1.23 | 6.33±0.69 |
| 多选题(Multiple choice questions) | 4.43±1.08 | 5.58±1.26 | 6.15±0.83 |
| 不及格(Fail (a test)) | 4.55±1.26 | 5.35±1.05 | 6.25±0.71 |
| 论述题(Essay questions) | 4.88±1.36 | 5.75±1.10 | 5.78±0.89 |
| 考试卷子(Examination paper) | 4.53±1.18 | 5.48±1.22 | 6.08±0.80 |
| 担心考试(Worried about the exam) | 4.65±1.35 | 5.35±0.92 | 6.15±0.70 |
| 期末考试(Final exam) | 4.55±1.32 | 5.23±1.14 | 6.10±0.78 |
| 考试名次(Examination ranking) | 4.53±1.38 | 5.40±0.87 | 6.13±0.88 |
| 考试成绩(Examination results) | 4.90±1.57 | 5.63±1.00 | 6.10±0.74 |
